# β1-adrenergic receptor polymorphisms: a possible genetic predictor of bisoprolol response in acute coronary syndrome

**DOI:** 10.2144/fsoa-2023-0113

**Published:** 2023-08-22

**Authors:** Mohamed S Fayed, Mohamed Ayman Saleh, Nagwa A Sabri, Amal A Elkholy

**Affiliations:** 1 Department of Clinical Pharmacy, Faculty of Pharmacy, Ain Shams University, Cairo, 11566, Egypt; 2 Department of Cardiology, Faculty of Medicine, Ain Shams University, Cairo, 1181, Egypt

**Keywords:** ADRB1, Arg389Gly, bisoprolol, blood pressure, heart rate, Ser49Gly

## Abstract

**Aim:**

To investigate the association between beta1-adrenergic receptor (*ADRB1*) polymorphisms and response to bisoprolol treatment in beta-blocker naive patients with acute coronary syndrome (ACS).

**Patients & methods:**

Seventy-seven patients received bisoprolol for four weeks. Blood pressure and heart rate were measured at baseline and during treatment. TaqMan allelic discrimination method was utilized for *ADRB1* Ser49Gly and Arg389Gly genotyping.

**Results:**

Arg389Arg carriers showed greater reductions in systolic and diastolic blood pressure (-8.5% ± 7.8% vs -0.76% ± 8.7%, p = 0.000218), and (-9.5% ± 9.7% vs -0.80% ± 11.5%, p = 0.000149), respectively, compared with Gly389 carriers. No statistical difference was found for study's outcomes based on codon 49.

**Conclusion:**

Arg389Gly polymorphism is a promising bisoprolol response predictor in ACS patients.

## Plain language summary

Pharmacogenetics is a field of study that explores how our genes can affect how well certain medicines work. In this study, scientists looked at a specific gene called beta1-adrenergic receptor to see how it can influence a drug called bisoprolol. They wanted to find out if some people's genes made bisoprolol work better for them.

They studied 77 patients with a heart problem called acute coronary syndrome (ACS) who were taking bisoprolol for 4 weeks. The researchers discovered that people with a particular gene piece called Arg389Arg responded better to bisoprolol. They had bigger reductions in their blood pressure compared with those who had a different gene called Gly389.

This finding suggests that by looking at a person's genes, doctors might be able to predict how well bisoprolol will work for them. This way, doctors can choose the best treatment for each patient, making sure they get the most benefit from the medicine.

## Tweetable abstract

Association between *ADRB1* polymorphisms & bisoprolol response in ACS patients: 77 patients received bisoprolol for 4 weeks. Arg389Arg carriers had greater blood pressure reduction compared with Gly389 carriers. Arg389Gly predicts bisoprolol response. #ACS #ADRB1 #Bisoprolol

## Graphical abstract

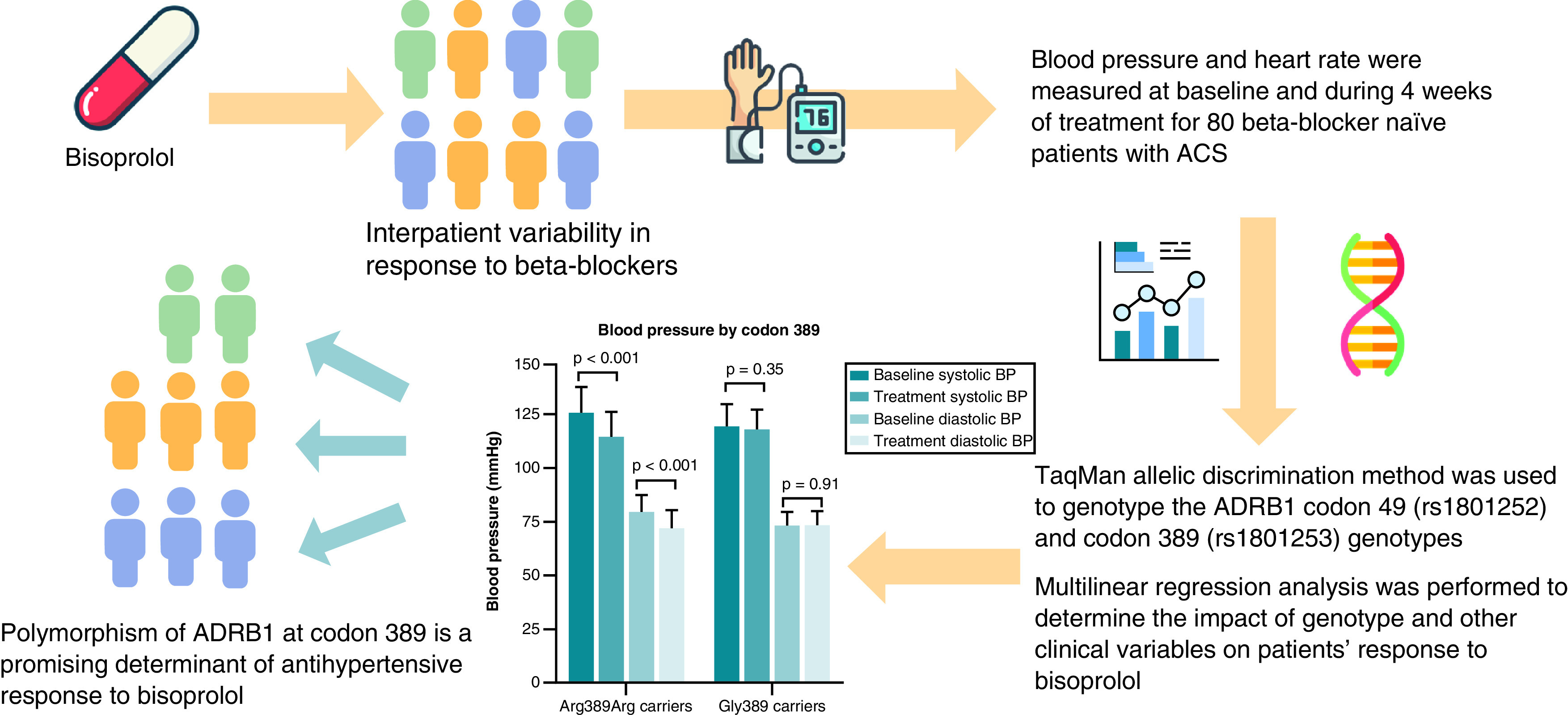

Cardio-selective beta1-receptor blockers, such as bisoprolol, are commonly used in patients with coronary artery disease (CAD) to reduce heart rate, contractility and blood pressure, thereby decreasing myocardial oxygen requirements [1], They also prolong filling time, enhancing coronary perfusion, and play a crucial role in treating acute coronary syndrome (ACS) [2,3].

Beta-1-Adrenergic receptor (ADRB1) is the primary target of beta-blockers, especially ADRB1-selective blockers like metoprolol, atenolol and bisoprolol. Genetic variants are frequently found in *ADRB1* at codons 49 ( rs1801252: Ser49Gly) and 389 (rs1801253: Arg389Gly), caused by single nucleotide polymorphisms (SNPs) of the *ADRB1* gene [4].

*In vitro* experiments have shown that these genetic polymorphisms alter the functionality of ADRB1. Regarding codon 389, data indicate that the Arg389 variant of the receptor displays basal and agonist-stimulated adenylyl cyclase activities 2- and threefold higher than the Gly389 variant. Hence, it is expected that patients with the Arg389Arg genotype will exhibit a better response to beta-blockers [5–7]. Functional data for codon 49 indicate that this polymorphism primarily impacts receptor regulation, with the Gly49 allele exhibiting greater agonist-mediated receptor down-regulation. Consequently, genotypes characterized by higher resistance to down-regulation, such as the Ser49Ser genotype, are anticipated to demonstrate enhanced responsiveness to beta-blockers [8,9].

Support for the influence of *ADRB1* polymorphisms on response to beta-blockers is available in cases of heart failure and hypertension [10,11]. However, the impact of these polymorphisms on blood pressure and heart rate response to beta blocker therapy in the treatment of ACS has received limited attention [12]. Available evidence has primarily focused on the association of *ADRB1* polymorphisms with mortality and the risk of developing ACS [13–16].

The significance lies in the fact that a modest prediction of a 2 mmHg antihypertensive response through pharmacogenetics can lead to a decreased long-term mortality risk associated with ischemic heart disease [17]. Furthermore, the majority of human studies examining the functional implications of these polymorphisms have primarily centered around metoprolol, atenolol and carvedilol [18–21]. However, the investigations on genetic variants influencing bisoprolol response are limited and perplexing [22]. Additionally, many of these studies failed to consider the potential impact of prior beta blocker exposure, leading to confounding factors due to the lingering antihypertensive effects [23].

Ethnic variations in the frequency of Ser49Gly and Arg389Gly polymorphisms have been documented in previous studies [24,25]. Moreover, interethnic disparities in the response to beta-blocker therapy have been observed, with African American patients showing a suboptimal response and Chinese patients exhibiting a more favorable response to beta-blockers [26,27]. These differential drug responses across ethnic groups have been linked to variations in the distribution of *ADRB1* polymorphisms. Notably, there is limited pharmacogenetic evidence available regarding the impact of *ADRB1* polymorphisms in the Egyptian population.

toward that end, the current study sought to investigate the association between *ADRB1* polymorphisms and the therapeutic effect of bisoprolol in beta-blocker naive Egyptian patients with ACS.

## Patients & methods

### Study design

This was a prospective cohort study conducted at Ain Shams University hospital, Cairo, Egypt, started from the first of May 2021 to the end of August 2021.

### Patient eligibility (inclusion & exclusion criteria)

The study included beta-blocker naive unrelated Egyptian patients aged between 30 and 75 years old with new onset of ACS. Patients with kidney or liver failure, malignancy, heart failure, pregnancy, systolic blood pressure (SBP) <90 mmHg at screening or heart rate (HR) <55 bpm based on resting ECG at screening were excluded from the study.

### Enrollment & allocation

Initially, 120 patients were screened for eligibility in the study. Out of this cohort, 40 patients were excluded for various reasons, including prior beta-blocker treatment, presence of heart failure, pregnancy, malignancy and refusal to participate. Consequently, the final study cohort consisted of 80 eligible patients who were included in the investigation.

### Data collection

Patients were interviewed upon admission to collect demographic data, including medical and medication history. Assessments of SBP, DBP, HR, kidney function tests, liver function tests, resting electrocardiogram and echocardiography were conducted at baseline, during treatment, and after 4 weeks. Patients were administered daily oral doses of bisoprolol fumarate tablets (Concor^®^) at either 2.5 mg or 5 mg for a duration of 4 weeks. Upon discharge, patients were instructed to measure their seated SBP, DBP, and HR on a daily basis, with readings taken in the morning, afternoon and evening. A minimum of 21 BP measurements were recorded prior to each visit to the cardiac rehabilitation clinic. Weekly follow-up visits were conducted at the clinic to assess office BP and HR, collect home BP and HR follow-up sheets, ensure compliance and document any intolerable side effects. Consistent scheduling of clinic visits at the same time of day minimized the impact of diurnal variation on BP measurements during treatment.

### DNA extraction & genotyping

Venous blood was obtained from each subject into 5-ml EDTA tube, genomic DNA was extracted from leukocytes with automated QIAcube device using QIAamp DNA Blood Mini Kit (Qiagen, Hilden, Germany) according to the manufacturer's guidelines. Polymorphisms were selected from candidate gene (*ADRB1*): (rs1801252 and rs1801253). The selected polymorphisms were genotyped by TaqMan allelic discrimination method using TaqMan genotyping probes (C_8898508_10, C_8898494_10, respectively), (Applied Biosystems, Thermo Fisher Scientific, MA, USA) and carried out by using real-time polymerase chain reaction with Rotor gene Q (Qiagen, Hilden, Germany) utilizing TaqMan Universal PCR Master Mix (Applied Biosystems^®^) in accordance with the manufacturer's instructions. Importantly, both the subjects and investigator were blinded to the genotypic results throughout the study period.

### Ethics

The study was conducted in accordance with the ethical principles outlined in the Declaration of Helsinki. Approval for the study was obtained from the ethics committee of the College of Pharmacy, Ain Shams University, Cairo, Egypt (approval number 261). Prior to participating in the study, all patients were informed about the study protocol, and written informed consent was obtained from each participant.

### Statistical analyses

Hardy-Weinberg equilibrium was assessed by Pearson's chi-square test. Comparisons of baseline demographic data and cardiovascular parameters among groups were performed by the chi-square test, Fisher's exact test for count and general linear model, as appropriate. The association between polymorphisms and response to bisoprolol was evaluated by general linear model. Statistical analyses were performed using IBM SPSS Statistics for Windows, Version 26.0.(Armonk, NY: IBM Corp). p < 0.05 was considered statistically significant. All continuous variable data are shown as the mean ± SD. Linear regression models were used to examine the joint effects of covariates on the change in SBP and DBP. The following variables were analyzed for inclusion in the model: baseline mean DBP or SBP, age, sex, codon 389 genotype and codon 49 genotype.

Because Gly49Gly and Gly389Gly genotypes were expected to occur infrequently in the study population, it was planned a priori that these homozygotes would be combined with heterozygotes and the groups classified as Gly49 carriers and Gly389 carriers for analysis.

### Sample size calculation

Johnson *et al.* [18] reported that Arg389 homozygotes showed larger decreases in 24 hour DBP in response to metoprolol than Gly389 homozygotes (-12% ± 8.6% vs -5.1% ± 7.8%). The power calculation using these data indicated that 31 subjects in each group would be required to detect the differences in DBP between genotypes of 6.9%, with a power of 90% with a 2-tailed α of 0.05. A total of 80 subjects were included in the study to compensate any subjects' withdrawal or non-adherence.

## Results

### Study enrollment & patients' allocation

**Figure 1.**
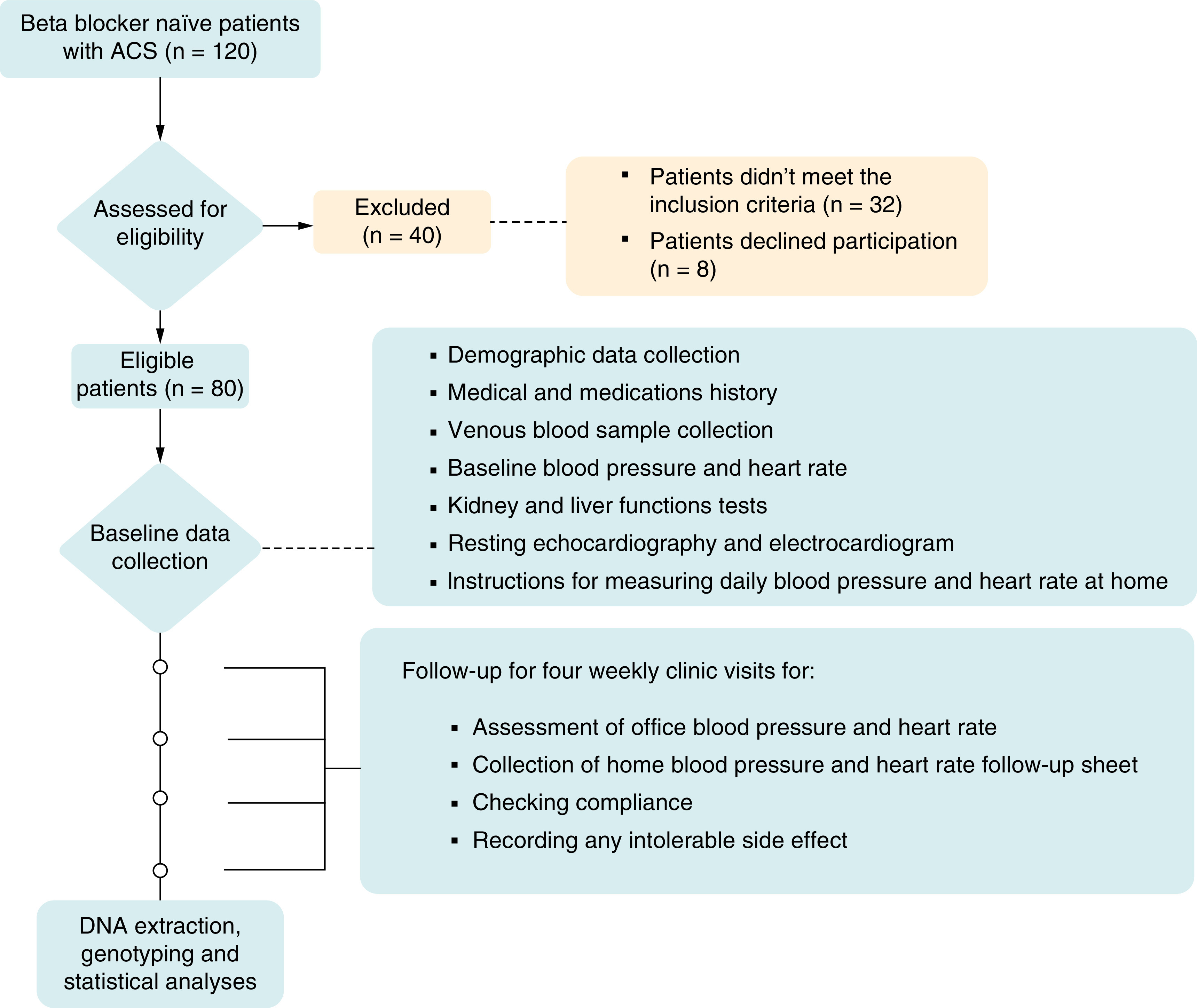
Study flowchart representing enrollment, allocation, follow-up and analysis processes.

As presented in Figure 1, One hundred and twenty beta-blocker-naive patients with ACS were assessed for eligibility. Out of these, 40 patients were excluded (32 for not meeting the inclusion criteria and 8 who chose not to participate), leaving 80 patients for prospective interviews. Ultimately, 77 subjects successfully completed the study, with three subjects withdrawing for personal reasons.

### Genotyping results

The observed allele frequencies did not deviate from the Hardy–Weinberg equilibrium expectations for codons 389 and 49 (p = 0.09 and p = 0.3, respectively). The minor allele frequencies (MAF) were 14% for the Gly-allele of rs1801252, and 36% for the Gly-allele of rs1801253 (Table 1).

**Table 1.** The genotypic frequencies of the studied SNPs.

SNP	Total (n = 77)	MAF	p-value
rs1801252, n (%)			0.3
*Ser/Ser*	56 (72.7%),		
*Ser/Gly*	21 (27.3%).	0.14	
*Gly/Gly*	0		
rs1801253, n (%)			
*Arg/Arg*	28 (36.4%)		0.09
*Arg/Gly*	43 (55.9%)	0.36	
*Gly/Gly*	6 (7.8%)		

p-value, using the χ2 test to test the deviation of genotype distribution from the predicted genotype frequencies based on the Hardy–Weinberg equilibrium.

MAF: Minor allele frequency; SNP: Single nucleotide polymorphism.

**Figure 2.**
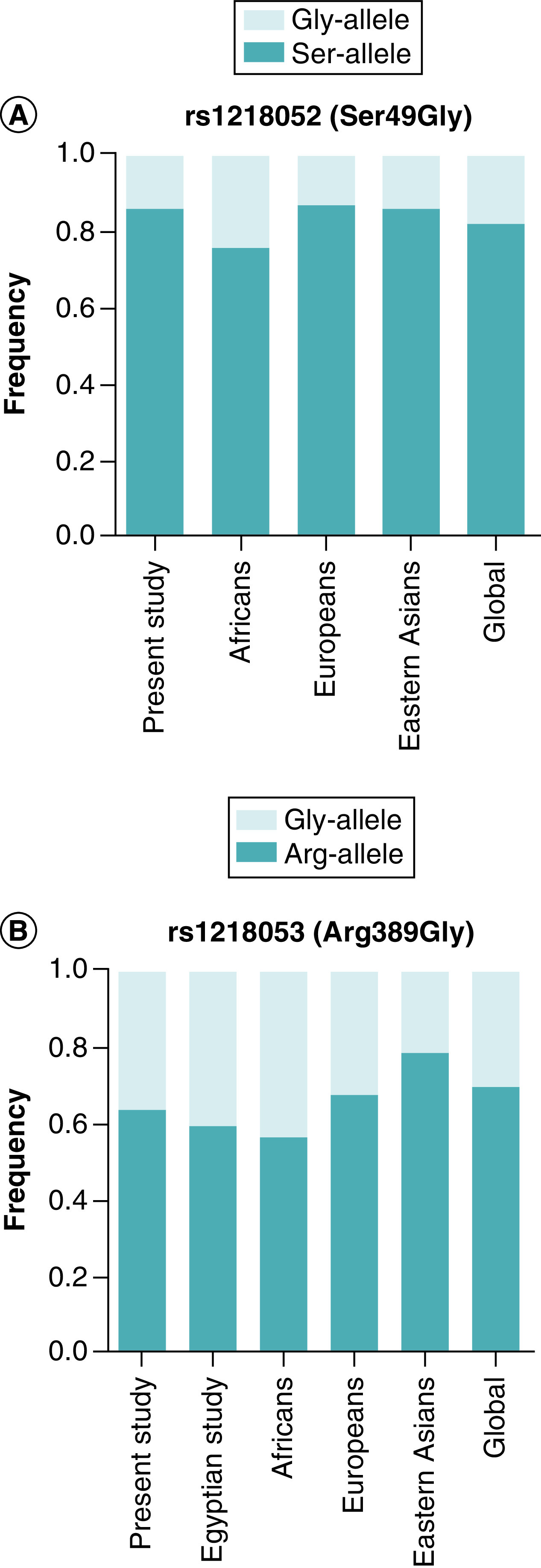
Comparison of allele frequencies of the studied SNPs in Egyptians compared with other populations. **(A)** *ADRB1* rs1801252 (light bars: Gly-allele, dark bars: Ser-allele), **(B)** *ADRB1* rs1801253 (light bars: Gly-allele, dark bars: Arg-allele). Allele frequencies obtained from the 1000 Genome, Phase III data [28]. Egyptian study, El Gindy *et al.* 2022 [29].

Comparing MAF of the studied SNPs in Egyptians with African, European Asian and global populations showed distinct patterns (Figure 2) [28]. The rs1801252 Gly-allele frequency was similar to Europeans, East Asians and the global population, but lower than Africans. The rs1801253 Gly-allele frequency was closer to Africans and a recent pharmacogenetic study in Egyptians [29] but higher than Europeans, Asians and global population.

Previous studies have documented varying degrees of linkage disequilibrium (LD), ranging from weak to strong, for these two *ADRB1* SNPs [30–32]. In our cohort, we observed weak LD between these two SNPs (D' = 0.574 and R^2^ = 0.029).

### Demographic & clinical characteristics

Baseline characteristics by codon 389 genotype and codon 49 genotype are shown in (Table 2). The age, sex, smoking habit and comorbidities did not differ between the Arg389 homozygotes and Gly389 carriers. However, baseline SBP and DBP were statistically different between the two groups, basal values for SBP were 125.50 ± 14.04 among Arg389 homozygotes and 119.39 ± 11.86 among Gly389 carriers (p < 0.05). Furthermore, basal values for DBP were 79.75 ± 8.71 among Arg389 homozygotes and 73.20 ± 7.19 among Gly389 carriers (p < 0.01). There was no statistically significant difference in baseline HR between the two groups. No statistically significant difference in the demographic and clinical characteristics were observed between Ser49 homozygotes and Gly49 carriers.

**Table 2.** Baseline characteristics by codon 389 genotype and codon 49 genotype.

Variable	Arg389 homozygotes (n = 28)	Gly389 carries (n = 49)	p-value	Ser49 homozygotes (n = 56)	Gly49 carriers (n = 21)	p-value
Age (years), mean (SD)	57.18 (11.18)	56.35 (9.00)	0.72^†^	56.34 (9.90)	57.48 (9.66)	0.65^†^
Male, n (%)	22 (78.6%)	44 (89.8%)	0.18^‡^	48 (85.7%)	18 (85.7%)	1.00^‡^
Ex-smoker, n (%)	13 (46.4%)	24 (49%)	0.83^‡^	28 (50%)	9 (42.86%)	0.58^‡^
Diabetes, n (%)	7 (25.0%)	18 (36.7%)	0.29^‡^	18 (32.1%)	7 (33.3%)	0.92^‡^
Hypertension, n (%)	9 (32.1%)	23 (46.9%)	0.21^‡^	24 (42.9%)	8 (38.1%)	0.71^‡^
Atrial fibrillation, n (%)	0 (0.0%)	4 (8.2%)	0.29^§^	2 (3.6%)	2 (9.5%)	0.30^§^
Baseline HR (beats/min), mean (SD)	85.89 (9.25)	83.10 (11.15)	0.27^†^	83.91 (10.85)	84.67 (9.84)	0.78^†^
Baseline systolic BP (mm Hg), mean (SD)	125.50 (14.04)	119.39(11.86)	**<0.05** ^†^	121.25 (12.58)	122.57 (14.15)	0.69^†^
Baseline diastolic BP (mm Hg), mean (SD)	79.75 (8.71)	73.20 (7.19)	**<0.01** ^†^	75.11 (8.51)	76.86 (7.96)	0.42^†^
Bisoprolol dose for 4 weeks, n (%)			0.69^‡^			0.47^‡^
5 mg	9 (32.2%)	18 (36.7%)		21 (37.5%)	6 (28.6%)	
2.5 mg	19 (67.8%)	31 (63.3%)		35 (62.5%)	15 (71.43%)	
Spironolactone, n (%)	2 (7%)	10 (20.4%)	0.19^§^	9 (7%)	3 (20.4%)	1.00^§^
Nitroglycerin, n (%)	7 (25%)	9 (18.4%)	0.49^‡^	14 (25%)	2 (9.5%)	0.21^§^
ACE inhibitors, n (%)	24 (85.7%)	40 (81.6%)	0.65^‡^	49 (87.5%)	40 (71.43%)	0.09^‡^
Loop diuretics, n (%)	2 (7%)	10 (20.4%)	0.19^§^	11 (19.64%)	1 (4.76%)	0.16^§^

† General linear model.

‡ Pearson's Chi-squared test.

§ Fisher's exact test.

Data are given as mean ± SD, Standard deviation where appropriate.

Bold values indicate statistical significance.

ACE: Angiotensin converting enzyme; BP: Blood pressure; HR: Heart rate.

### Medications history

The majority of patients were prescribed angiotensin-converting enzyme inhibitors (83%), with smaller proportions receiving nitroglycerin (20.8%), spironolactone (15.6%) and loop diuretics (15.6%). Furthermore, all patients were taking clopidogrel, aspirin and high-intensity statins. The allocation of medications, particularly for blood pressure-lowering drugs, was consistent among all genotypes (Table 2).

### Safety & tolerability

Adverse effects from bisoprolol in this population were minor (headache, dizziness and diarrhea). None of the study participants discontinued the study because of adverse effects.

### Effect of bisoprolol on blood pressure & heart rate

**Figure 3.**
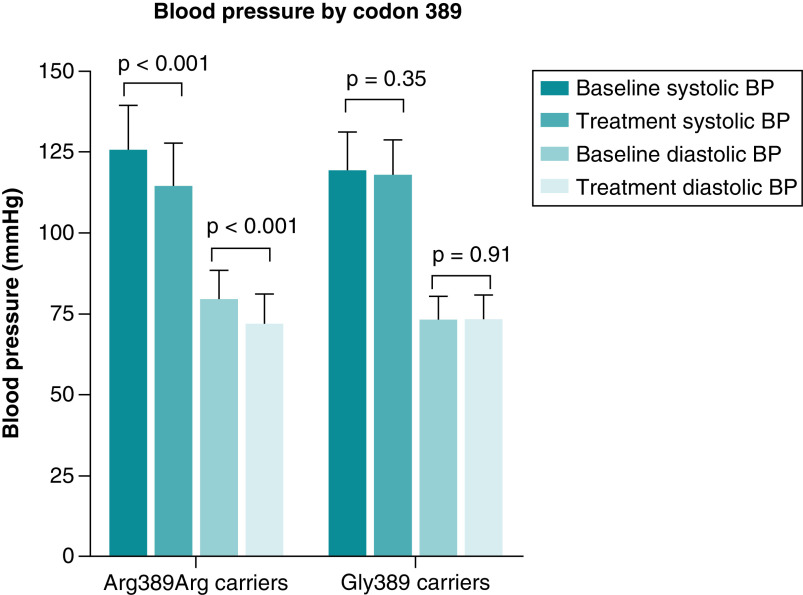
Bar chart presenting the changes in systolic and diastolic blood pressure after 4 weeks of bisoprolol treatment according to codon 389 genotype. P-values were computed from paired *t*-test between baseline (before treatment) and average BP readings during 4 weeks of treatment (post treatment). Showing a significant change of both systolic and diastolic blood pressure from baseline among the Arg389Arg carriers (p < 0.001 and p < 0.001, respectively), inconsistent with Gly389 carriers which showed no significant changer neither in systolic (p = 0.35) nor diastolic (p = 0.91) blood pressure. Data are presented as mean with SD Created by GraphPad Prism version 8.0.2 for Windows. BP: Blood pressure; mmHg: Millimeters of mercury.

As shown in Figure 3, SBP and DBP responses to bisoprolol were significantly different between codon 389 genotype groups. After 4 weeks of treatment with bisoprolol, only Agr389 homozygotes showed a significant change in both systolic (p < 0.001) and diastolic (p < 0.001) blood pressure from baseline.

**Figure 4.**
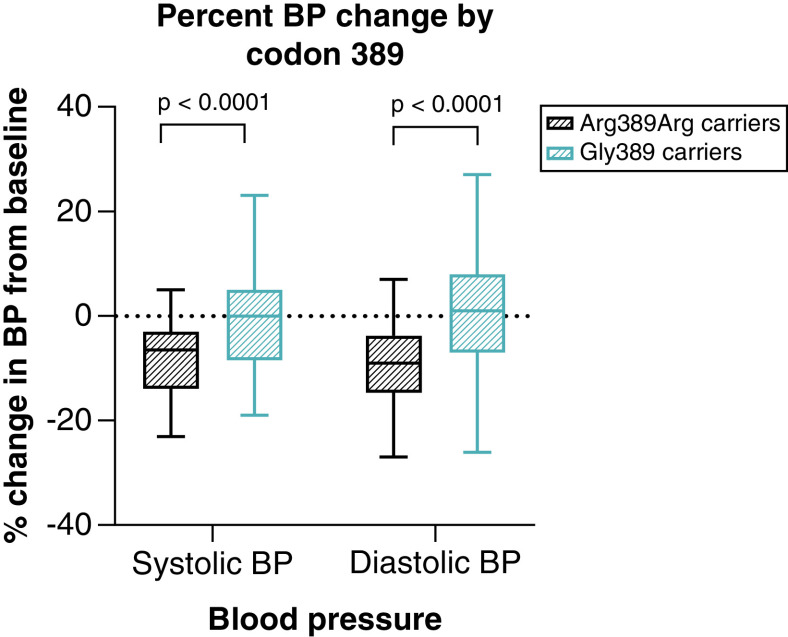
Boxplot presenting the percent change in blood pressure compared with baseline in response to bisoprolol by codon 389 genotype. P-values were computed from the general linear model. Showing a significant difference in percent change in blood pressure between Arg389Arg carriers and Gly389 carriers (p < 0.0001). Created by GraphPad Prism version 8.0.2 for Windows.

In Arg389 homozygotes, the reduction in DBP was remarkably greater than that in Gly389 carriers (-9.5% ± 9.7% vs -0.80% ± 11.5%, p = 0.00015), representing an absolute reduction in DBP that was 8 mm Hg greater than that in Gly389 carriers (95% confidence interval, -8.8 to -7.3 mm Hg; p = 0.00012). There was also a greater reduction in SBP in the Arg389 homozygotes compared with Gly389 carriers (-8.5% ± 7.8% vs -0.76% ± 8.7%, p = 0.00022), representing an absolute reduction in SBP that was -9.6 mm Hg greater than that in Gly389 carriers (95% confidence interval, -10.5 to -8.7 mm Hg; p = 0.00012) (Figure 4).

**Figure 5.**
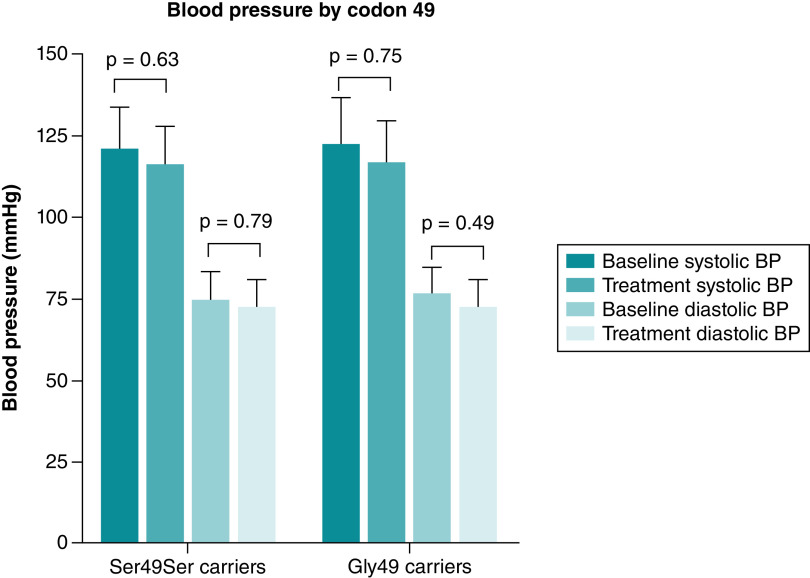
Bar chart presenting the systolic and diastolic blood pressure changes after 4 weeks of bisoprolol treatment according to codon 49 genotype. P-values are computed from paired t test between baseline (before treatment) and average BP readings during 4 weeks of treatment (post treatment). BP, blood pressure; mmHg, millimeters of mercury. Data are presented as mean with SD. Created by GraphPad Prism version 8.0.2 for Windows.

As shown in Figure 5, systolic and diastolic blood pressure responses to bisoprolol were not significantly different between codon 49 genotype groups.

**Figure 6.**
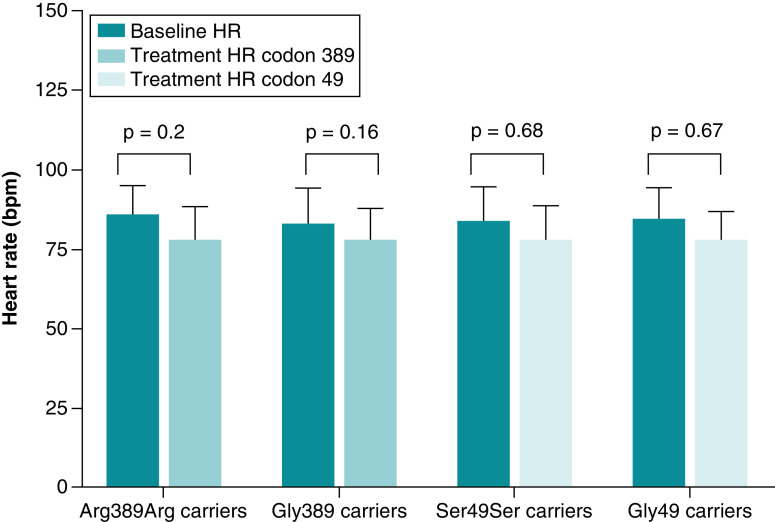
Heart rate changes after 4 weeks of bisoprolol treatment according to codon 389 genotype. The p-values are computed from paired *t*-test between baseline and week 4. Data are presented as mean with SD. Created by GraphPad Prism version 8.0.2 for Windows. bpm: Beat per minute; HR: Heart rate.

On the other hand, there were no differences in the HR response to bisoprolol based on the two studied codons (Figure 6).

### Multivariate analysis

Linear regression analysis was used to examine the effects of multiple variables on the change in DBP and SBP. Of the five variables considered for inclusion in the model, only baseline mean DBP, codon 389 genotype were significant predictors of the change in DBP (adjusted R^2^ = 0.34, p < .0001). Likewise, only baseline mean SBP, codon 389 genotype were significant predictors of the change in SBP (adjusted R^2^ = 0.33, p < 0.0001) (Table 3). The model suggests significant independent SBP-lowering and DBP-lowering contributions of -7.2 mm Hg and -4.7 mmHg associated with the Arg389Arg genotype, respectively.

**Table 3.** Factors influencing the change in blood pressure in response to administration of bisoprolol in multivariate analysis.

Predictors	Change in DBP		Change in SBP	
	Parameter estimate	p-value	*Parameter estimate*	p-value
Baseline DBP	‐0.52	**<0.001**	–	–
Baseline SBP	–	–	‐0.41	**<0.001**
Sex (male)	‐1.42	0.566	0.519	0.868
Age	‐0.07	0.428	0.07	0.525
Arg389Arg genotype	‐4.68	**0.018**	‐7.214	**0.002**
Ser49Ser genotype	0.09	0.963	0.925	0.701
Intercept	39.05		35.195	
R^2^ adjusted	0.34		0.33	

Expected change in DBP (millimeters of mercury) = 39.05 - 0.52(Baseline DBP) -1.42(if male) – 0.07(Age) + 4.68 (if Arg389Arg) + 0.09 (if Ser49Ser). Expected change in SBP (millimeters of mercury) = 35.195 – 0.41(Baseline SBP) + 0.519 (if male) – 0.07(Age) + 7.214 (if Arg389Arg) + 0.925 (if Ser49Ser).

Bold values indicate statistical significance.

DBP: Diastolic blood pressure; SBP: Systolic blood pressure.

## Discussion

Pharmacogenetics aims to enable personalized medicine by recognizing the impact of genetic variations on individual responses to medications. Variations in genes can influence receptor sensitivity or drug metabolism, potentially explaining these discrepancies [33]. The effectiveness of beta-blockers primarily relies on their interaction with ADRB1, a key regulator of the sympathetic nervous system [34,35]. However, individual responses to beta-blockers differ, possibly due to *ADRB1* gene polymorphisms [36,37].

The frequency of the rs1801252 Gly-allele (Ser49Gly polymorphism) in our study was found to be 14% which is close to the frequency of Europeans (13%), similar to East Asians (14%) but lower than Africans (24%), importantly, this's the first study to provide data on allelic frequency for Ser49Gly polymorphism in the Egyptian population. On the other hand, the frequency of the rs1801253 Gly-allele (Arg389Gly) was found to be 36% which is in good alignment with the findings of recent pharmacogenetic study in Egyptians (40%) [29], to some degree different from Africans (42%) and Europeans (32%) but higher than East Asians (21%). The discrepancy in the MAF of the studied SNPs between Egyptians and other populations implies a potential admixed genetic makeup among Egyptians. Further pharmacogenetic studies are needed to gain a deeper understanding of the ethnic diversity within the Egyptian population.

Multiple studies have linked *ADRB1* polymorphisms to the antihypertensive response of beta-blockers like metoprolol and carvedilol [37]. However, there is limited information on the association with bisoprolol [38–44], especially in the Egyptian population. To our knowledge, this is the first study examining the potential impact of the Arg389Gly and Ser49Gly polymorphisms on bisoprolol response in Egyptians.

For codon 389, whereas some studies failed to find significant effects of *ADRB1* gene polymorphisms on blood pressure responses to beta-blockers [45–48], others have reported a substantial contribution for this polymorphism in explaining some of the variability in response toward different beta-blockers [18–21,38,41,43,49]. We found that the Arg389 homozygous genotype was associated with better SBP and DBP response to bisoprolol. This finding was consistent across every type of analysis conducted. Furthermore, the Arg389Arg genotype was a significant predictor of change in SBP and DBP in multivariate analysis.

Apart from some studies that demonstrated a greater response to beta-blockers among the Gly389 carriers [41,43,49], our study is showing the same direction of association, namely, Arg389 homozygotes had the greatest blood pressure response with most of published studies on the impact of this polymorphism [18–21,38,50]. Johnson *et al.* [18], for instance, found that Arg389Arg carriers had a nearly threefold greater reduction in daytime diastolic blood pressure (-13.3% ± 8.4% vs -4.5% ± 8.2%, p = .0018) compared with Gly389 carriers. Liu *et al.* [20] confirmed the results of Johnson *et al.*, in an open trial with 61 hypertensive Han Chinese, showing best response ( in both SBP and DBP) to 4 weeks of treatment of metoprolol in patients homozygous for the Arg389 carriers. Consistently, Dayong Si *et al.* [21] showed that hypertensive patients homozygous for *ADRB1* Arg389 had an approximately fourfold greater reduction in DBP than those homozygous for *ADRB1* Gly389 (10.61 vs 2.62 mm Hg, p = 0.013). In addition, there are two other small studies with healthy volunteers suggesting better response to beta blockers with Arg389 carriers [19,51].

Deciphering the incongruent findings among pharmacogenomics studies presents a notable challenge. Various factors may contribute to the disparities in results between our study and others. Firstly, a notable distinction is that our study adopted a prospective design, while many negative studies were retrospective, relying on pharmacogenetic associations tested on existing databases. Retrospective designs are more prone to confounding and biases, which might contribute to the disparities in findings [36].

Secondly, the minute-to-minute variability in BP and HR presents challenges in obtaining precise phenotype data. Variations in methodologies used to measure these variables among studies can lead to differing observations on the effects of *ADRB1* genotypes or even impede the ability to distinguish the impact of the studied polymorphisms. For instance, Filigheddu *et al.* [46] examined the response to atenolol in hypertensive patients based on *ADRB1* polymorphisms. They recorded office BP and HR values every 2 weeks and analyzed data only of week 4 and 8, while other studies used a more controlled approach like ambulatory blood pressure monitoring [18,41]. In our study, we took precautions to minimize these effects by collecting both home and office measurements, ensuring compliance through weekly visits, and consistently scheduling clinic visits at the same time of day to mitigate the influence of diurnal variation on BP measurements during treatment.

Additionally, it is crucial to consider differences in study inclusion and exclusion criteria, as well as the underlying pathophysiological mechanisms of the targeted population's diseases. Our study enrolled patients with ACS, in contrast to previous studies that focused on essential hypertension [20,21,45–47], heart failure [39,42,43], atrial fibrillation [32] or healthy volunteers [19,48,51]. Furthermore, we specifically included beta-blocker-naive patients to eliminate any lingering effects of previous exposure to beta-blockers.

Moreover, the results of previous studies suggest that the therapeutic impact of the *ADRB1* genotype differs according to selectivity and dose of beta-blockers. Notably, Parikh *et al.* [52] demonstrated in a retrospective analysis of prospectively designed DNA sub-studies from Beta-Blocker Evaluation of Survival Trial (BEST) [53] and Heart Failure: A Controlled Trial Investigating Outcomes of Exercise Training (HF-ACTION) trial [54] that enhanced efficacy of bucindolol in heart failure with reduced ejection fraction occurs at high doses for individuals with the *ADRB1* Arg389Arg genotype compared with Gly389 carriers. Lastly, the lack of pharmacokinetic assessment may also have contributed to the observed disparities in the findings.

In addition to the significant association of codon 389 with bisoprolol response, it was found that baseline systolic and diastolic blood pressure were significantly higher in Arg389 homozygotes than that in Gly389 carriers, this interesting finding is in good alignment with results seen in meta-analysis that included 86588 Individuals [55]. Genome-wide association study (GWAS) using electronic health records identified Arg389Gly as considerable locus for variation in SBP and DBP, reaching GWAS significance level ( p = 2 × 10^-10^ for SBP and 4 × 10^-12^ for DBP) [56], this was also evident in a large-scale multi-ancestry GWAS accounting for smoking behavior, Arg389Gly was found to be a significant variant for both SBP (p = 1.3 × 10^-22)^ and DBP (p = 3 × 10^-22^) [57], these findings from GWAS are of great value, as they represent a more unbiased way to assess the association of a certain polymorphism with a specific trait.

Regarding HR, in line with the majority of studies investigating the association between Arg389Gly polymorphism and HR response to beta-blockers, which provided no evidence of such association [18,20,37,39,42,43,58]; the present study found no difference in HR response between Arg389 homozygotes and Gly389 carriers.

The specific molecular mechanisms underlying the interactions between the studied polymorphisms and HR response remain unknown. However, several potential explanations for the lack of significant effects on HR response in this study can be proposed. One possibility is the modest dosage of bisoprolol used in our study (2.5 or 5 mg), which might not have fully blocked the more active *ADRB1* in Arg389Arg carriers. Consequently, it cannot be ruled out that different results might have been observed with a higher dose leading to a more substantial reduction in HR in the Arg389Arg genotype.

Another important consideration is that we did not assess the degree of diabetic cardiovascular autonomic neuropathy in the diabetic patients enrolled, representing 32.5% of our sample population. If significant diabetic autonomic dysfunction was present, HR response to bisoprolol might have been blunted in these individuals [59].

Furthermore, previous study by Rau *et al.* [42], revealed that Arg389Gly polymorphism significantly affected the heart rate-lowering effect of carvedilol but not bisoprolol in heart failure patients, particularly in those with atrial fibrillation (AF). These findings were corroborated by another study [32], suggesting a crucial role of the Arg389Gly polymorphism in heart rate control among AF patients treated with carvedilol.

Evident associations found with codon 389 in this study might be explained by the findings seen *in vitro* studies, in which the Arg389 allele showed three-times greater adenylyl cyclase activity in response to the agonist than those with the Gly389 allele, indicating that the abnormally active Arg389 receptor is more sensitive to pharmacological beta-blockade. From the opposing perspective, it might be construed that the Gly389 form of ADRB1 functions as if it were already blocked. Thus ADRB1 hyperactivity conferred by Arg at position 389 may represent an opportunity to use beta-blockers with greater effect in diseases responsive to antiadrenergic therapy [35].

Although the functional data for codon 49 suggest that the effect of this polymorphism is primarily on receptor regulation, with the Gly49 allele undergoing greater agonist-mediated receptor down-regulation, indicating a potential enhanced response to beta-blockers in the Ser 49 homozygotes [35], few studies have demonstrated only a non-significant tendency or minor contribution of the Ser49Gly polymorphism to the antihypertensive effect of beta-blockers [18,20,41]. Consistent with the available evidence [37,46–48,60], our study also found no statistically significant differences in any of the outcomes based on this codon.

The findings of our study should be interpreted in light of certain limitations. Firstly, we did not assess the impact of circulating markers such as sympathetic activation or renin-angiotensin system activity, particularly plasma renin activity, which has been suggested as a predictor of blood pressure response for certain medications [61,62]. However, there have been contradictory results in other reports [44,63,64]. Secondly, the generalizability of our findings to women may be limited due to the high proportion of men in the study population. Additionally, relying solely on blood pressure and heart rate measurements is insufficient to fully understand the influence of the studied genotypes on the bisoprolol outcomes in ACS patients. Evaluation of cardiac function and long-term follow-up including re-hospitalization and mortality should also be considered. Furthermore, the relatively low bisoprolol doses used in our study might have restricted the full pharmacogenetic effect. Therefore, it is plausible that different results could have been observed with higher doses, especially concerning heart rate response. Last, this study is constrained by its small sample size, potentially resulting in limited statistical power to detect significant differences concerning Ser49Gly polymorphisms, particularly considering its lower MAF compared with Arg389Gly. As this is the first study reporting the Ser49Gly MAF in the Egyptian population, future pharmacogenetic investigations targeting this population can utilize our data to calculate an appropriate sample size. Additionally, it is important to note that the sample size calculation was specifically focused on BP changes and may also be underpowered for assessing HR responses.

However, our study had a prospective design and only included beta-blocker naive subjects which provided a reliable results and eliminated the possible background noise due to persisting anti-hypertensive effect by the previous exposure to beta-blockers [23].

## Conclusion

The study's findings address the lack of data on allelic frequencies and ethnic origins in the Egyptian population for the studied SNPs. *ADRB1* Arg389Arg carriers exhibited a stronger response to bisoprolol in terms of changes in SBP and DBP, while no significant difference was observed for HR. Moreover, the Arg389Gly variant showed significance for both baseline SBP and DBP. The results of the present study support the consistency of pharmacogenetic findings across a variety of beta-blockers within the drug class. Therefore, the findings observed with bisoprolol in this study are likely to be applicable to other beta-blockers with similar characteristics, such as metoprolol and atenolol.

To validate these findings, future studies with larger cohorts are recommended. These studies should also explore the impact of haplotypes and multilocus genotypes and consider utilizing 24-h ambulatory BP monitoring for more accurate and reproducible data compared with clinic and home measurements.

**Summary points**

- Beta-1-Adrenergic receptor (*ADRB1*) gene polymorphisms at codons 49 and 389 have the potential to impact individual responses to beta-blockers. Previous studies have primarily focused on metoprolol, atenolol and carvedilol, with limited research on bisoprolol. The influence of *ADRB1* polymorphisms on blood pressure and heart rate response to beta-blocker therapy in the treatment of acute coronary syndrome (ACS) has received limited attention, particularly in the Egyptian population.
- This study therefore investigated the association between *ADRB1* polymorphisms and the therapeutic effect of bisoprolol in beta-blocker naive Egyptian patients with ACS, providing valuable insights into this population.
- *ADRB1* Arg389Arg carriers exhibited a stronger response to bisoprolol in terms of changes in systolic blood pressure (SBP) and diastolic blood pressure (DBP), while no significant difference was observed for heart rate (HR). The Arg389Gly variant showed significance for both baseline SBP and DBP.
- No statistically significant differences were found in any of the outcomes based on codon 49 (rs1801252: Ser49Gly) in this study.
- The study's findings addressed the lack of data on allelic frequencies and ethnic origins of the studied single nucleotide polymorphisms (SNPs) in the Egyptian population, particularly regarding the Ser49Gly polymorphism (rs1801252).
- Further studies with larger cohorts are needed to validate these findings and explore other factors such as haplotypes and multilocus genotypes.
